# Supplementary material for: MHC matching improves engraftment of iPSC-derived neurons in non-human primates
Source: Nat Commun. 2017 Aug 30;8:385. doi: 10.1038/s41467-017-00926-5 (PMC5577234; doi:10.1038/s41467-017-00926-5)
Supplement: Supplementary file 1 — Supplementary Information [file 41467_2017_926_MOESM1_ESM.pdf]

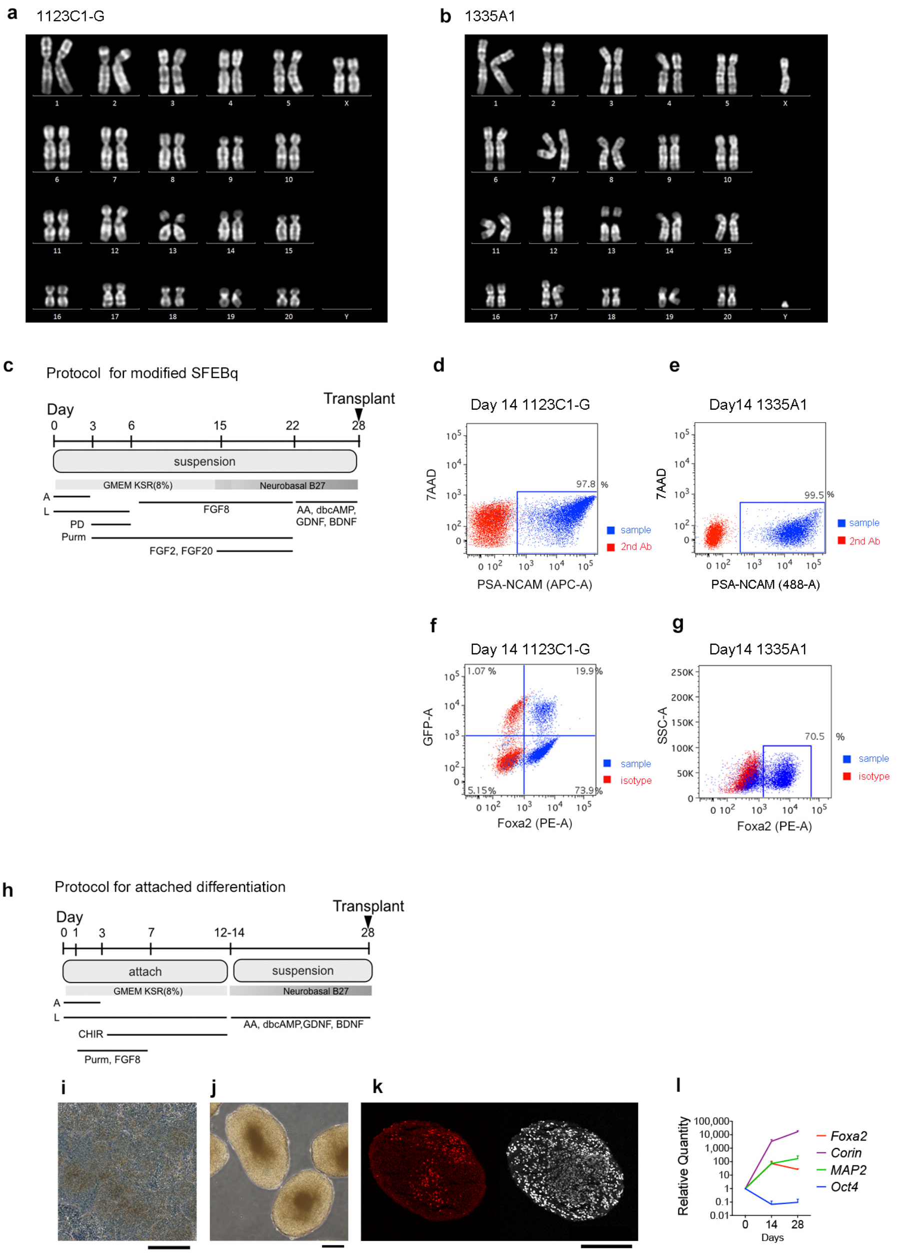

Supplementary Figure 1| Characterization of donor neurons

a, b, Karyotype analyses of HT1 (1123C1-G, a) and HT4 (1335A1, b) homozygous iPSC lines. 20 metaphases were analyzed and showed normal karyotype (42, XX for HT1, and 42 XY for HT4). **c-g,** Differentiation through modified SFEBq for Cont#1, #2, #5-#8, and Hetero#1, #2, #5-#8. Flow cytometric analyses showed that the

majority of differentiated cells was already positive for PSA-NCAM and Foxa2 at day14. Green fluorescent protein (GFP), a marker of donor cells for 1123C1-G, was positive in about 20% of the cells (f). (See also Fig. 2 g-o). h-l, Differentiation through the attached culture for Cont#3, #4, Hetero#3, and #4. For the first two weeks the cells were cultured as monolayer on iMatrix-coated plates (i), then the cells were detached and switched to sphere culture (j). Phase contrast pictures (i, j) and immunostaining of sliced spheres at day 28 (k, Nurr1; red, Foxa2; white) are shown. Scale bars: 200 μm. l, Quantitative PCR analysis of the cells during differentiation. GMEM: Glasgow MEM, KSR: Knockout serum replacement, A: A-83-01 (500 nM), L: LDN 193189 (100 nM), PD: PD0325901 (500 nM), CHIR: CHIR99021 (3 μM), Purm: Purmorphamine (2 μM), FGF2, 8, and 20; fibroblast growth factor 2 (5 ng/mL), 8 (100 ng/mL), and 20 (1 ng/mL), AA: ascorbic acid (200 μM), dbcAMP (400 μM), GDNF: glial cell line-derived neurotrophic factor (2 ng/mL), BDNF: brain-derived neurotrophic factor (20 ng/mL).

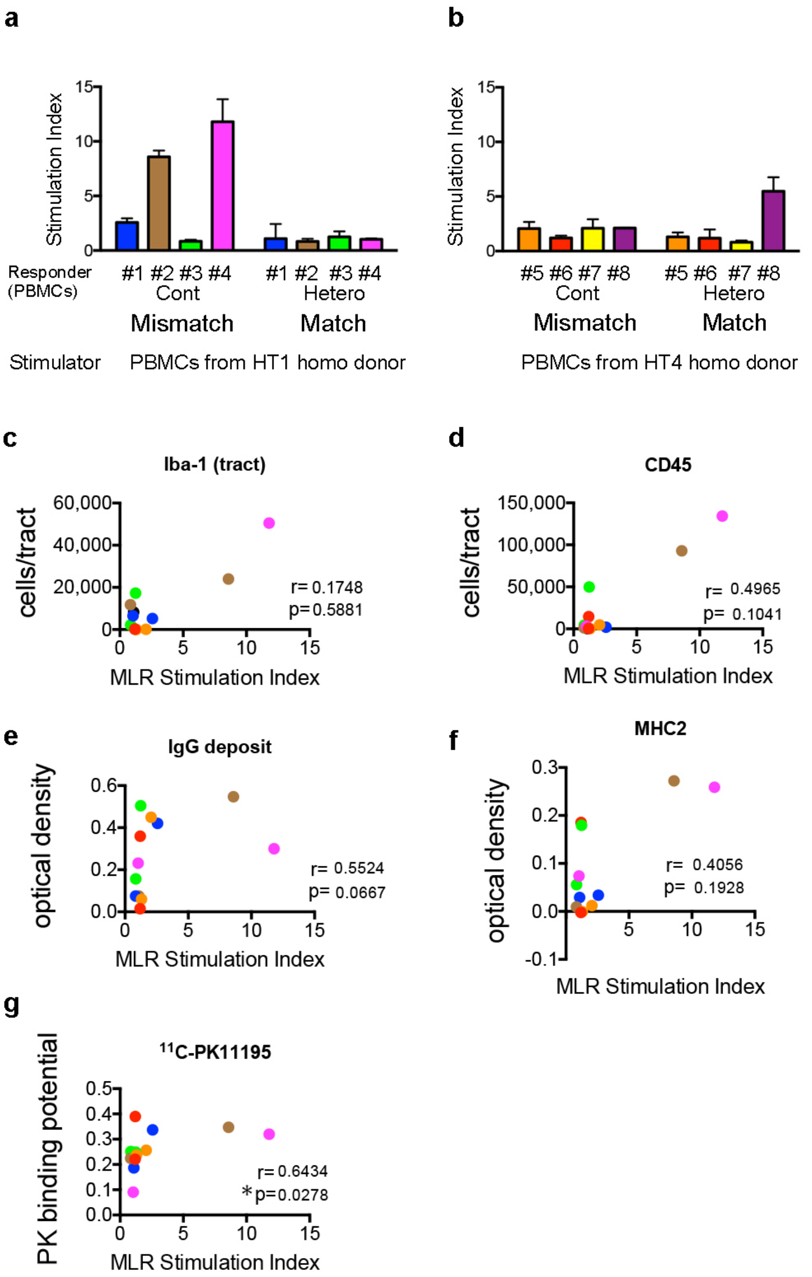

Supplementary Figure 2| Preoperative mixed lymphocyte reaction (MLR) and correlation to histological results and PET

a, b, One-way MLR between donor PBMCs as the stimulator and PBMCs from the hosts as the responders. Data are shown as the stimulation index (means ± SEM, n=3). c-g, Results from Spearman correlation analysis are given as r and p values in each graph.

Exepriment with the donor cells with HT1 homozygote

	Donor (DrpZ10-18B-B) Female	
	allele 1	allele 2
Mafa-F	F-like4	F-like4
Mafa-A	A1*052:02<br>A4*01:04<br>A8*01:01	A1*052:02<br>A4*01:04
Mafa-E	E-like5<br>E-like11	E-like5<br>E-like11
Mafa-B	B*095:01<br>B*033:02<br>B*098:10	B*095:01<br>B*033:02<br>B*098:10
Mafa-I	I*01:11	I*01:11
Mafa-DRB	DRB1*10:07<br>DRB1*03:21	DRB1*10:07<br>DRB1*03:21
Mafa-DQA1	DQA1*01:07:01	DQA1*01:07:01
Mafa-DQB1	DQB1*06:08	DQB1*06:08
Mafa-DPA1	DPA1*02:05	DPA1*02:05
Mafa-DPB1	DPB1*15:04	DPB1*15:04

	Cont#1 (DrpL5-5C-H) Male	
	allele 1	allele 2
Mafa-F	F-like1	F-like2
Mafa-A	A1*008:02<br>A1*086:02	A1*089:03<br>A2*05:50<br>A3*13:03:01
Mafa-E	E-like1	E-like10
Mafa-B	B*056:02:01<br>B*017:01<br>B*157:01<br>B*050:08<br>B*060:03:01<br>B*116:01<br>B*089:01:02	B*091:02<br>B*068:08
Mafa-I	I*01:01:01<br>I*01:11	I*01:14
Mafa-DRB	DRB*W53:01<br>DRB*W54:01	DRB1*W33:02
Mafa-DQA1	DQA1*01:18	DQA1*24:11
Mafa-DQB1	DQB1*06:35	DQB1*18:13
Mafa-DPA1	DPA1*04:02	DPA1*02:26
Mafa-DPB1	DPB1*03:04	DPB1*08:03

	Cont#2 (DrpZ3-2C-A) Male	
	allele 1	allele 2
Mafa-F	F-like4	F-like7
Mafa-A	A1*071:02<br>A4*14:14<br>A8*01:01	A1*093:01<br>A1*074:02
Mafa-E	E-like1	E-like9
Mafa-B	B*160:01<br>B*007:01:02<br>B*070:05<br>B*115:04:02	B*160:01<br>B*007:01:02<br>B*070:05<br>B*115:04:02
Mafa-I	I*01:11	I*01:11
Mafa-DRB	DRB*W1:04	DRB1*W36:01<br>DRB1*W1:08
Mafa-DQA1	DQA1*01:13	DQA1*26:03
Mafa-DQB1	DQB1*06:17:02	DQB1*06:17:02
Mafa-DPA1	DPA1*07:04	DPA1*02:15:02
Mafa-DPB1	DPB1*21:01	DPB1*10:01

	Cont#3 (DrpZ9-31C-A) Male	
	allele 1	allele 2
Mafa-F	F-like2	F-like2
Mafa-A	A1*089:03<br>A2*05:50<br>A3*13:03:01	A1*008:02<br>A1*086:02
Mafa-E	E-like3_New<br>E-like10	E-like9
Mafa-B	B*056:02:01<br>B*017:01<br>B*157:01<br>B*050:08<br>B*060:03:01<br>B*116:01<br>B*089:01:02	B*137:03<br>B*033:02
Mafa-I	I*01:01:01<br>I*01:11	I*01:18:02
Mafa-DRB	DRB*W53:01<br>DRB*W54:01	DRB*W53:01<br>DRB*W54:01
Mafa-DQA1	DQA1*01:18	DQA1*01:18
Mafa-DQB1	DQB1*06:35	DQB1*06:35
Mafa-DPA1	DPA1*04:02	DPA1*04:02
Mafa-DPB1	DPB1*03:04	DPB1*03:04

	Cont#4 (DrpZ10-43B-B) Male	
	allele 1	allele 2
Mafa-F	F-like1	F-like4
Mafa-A	A1*089:03<br>A2*05:50<br>A3*13:03:01	A1*098:02<br>A2*24:06
Mafa-E	E-like3<br>E-like10	E-like3<br>E-like10
Mafa-B	B*104:03<br>B*144:03N<br>B*057:04<br>B*060:02<br>B*046:01:02<br>B*050:08<br>B*114:02<br>B*072:01	B*159:01<br>B*007:01:01<br>B*158:01<br>B*085:01<br>B*079:02:02<br>B*098:09
Mafa-I	I*01:12:01	I*01:13:01
Mafa-DRB	DRB*W36:01<br>DRB*W3:01<br>DRB*W1:08	DRB1*03:19<br>DRB*W33:04
Mafa-DQA1	DQA1*26:03	DQA1*24:05
Mafa-DQB1	DQB1*18:07:02	DQB1*18:05
Mafa-DPA1	DPA1*02:15:02	DPA1*07:04
Mafa-DPB1	DPB1*10:01	DPB1*21:01

	Hetero#1 (DrpZ7-32B-B) Male	
	allele 1	allele 2
Mafa-F	F-like4	F-like4
Mafa-A	A1*052:02<br>A4*01:04	A1*094:01
Mafa-E	E-like5<br>E-like11	E-like1
Mafa-B	B*095:01<br>B*033:02<br>B*098:10	B*095:01<br>B*033:02<br>B*098:10
Mafa-I	I*01:11	I*01:11
Mafa-DRB	DRB1*10:07<br>DRB1*03:21	DRB1*10:06<br>DRB1*03:07
Mafa-DQA1	DQA1*01:07:01	DQA1*01:20
Mafa-DQB1	DQB1*06:08	DQB1*06:25
Mafa-DPA1	DPA1*02:05	DPA1*07:02
Mafa-DPB1	DPB1*15:04	DPB1*19:03

	Hetero#2 (2DrpZ4-33C-A) Male	
	allele 1	allele 2
Mafa-F	F-like4	F-like1
Mafa-A	A1*052:02<br>A4*01:04	A1*089:03<br>A2*05:50<br>A3*13:03:01
Mafa-E	E-like5<br>E-like11	E-like10
Mafa-B	B*095:01<br>B*033:02<br>B*098:10	B*099:01<br>B*108:01<br>B*045:05<br>B*050:08<br>B*114:02<br>B*072:01
Mafa-I	I*01:11	I*01:12:01
Mafa-DRB	DRB1*10:07<br>DRB1*03:21	DRB*W3:01<br>DRB*W36:01<br>DRB*W1:08
Mafa-DQA1	DQA1*01:07:01	DQA1*26:03
Mafa-DQB1	DQB1*06:08	DQB1*18:07:02
Mafa-DPA1	DPA1*02:05	DPA1*07:04
Mafa-DPB1	DPB1*15:04	DPB1*21:01

	Hetero#3 (DrpZ3-50C-A) Male	
	allele 1	allele 2
Mafa-F	F-like4	F-like4
Mafa-A	A1*052:02<br>A4*01:04	A1*018:04<br>A3*13:03:01
Mafa-E	E-like5<br>E-like11	E-like3
Mafa-B	B*095:01<br>B*033:02<br>B*098:10	B*099:01<br>B*108:01<br>B*045:05<br>B*098:09
Mafa-I	I*01:11	I*01:10:02
Mafa-DRB	DRB1*10:07<br>DRB1*03:21	DRB1*10:07<br>DRB1*03:21
Mafa-DQA1	DQA1*01:07:01	DQA1*26:03
Mafa-DQB1	DQB1*06:08	DQB1*18:07:02
Mafa-DPA1	DPA1*02:05	DPA1*04:02
Mafa-DPB1	DPB1*15:04	DPB1*03:04

	Hetero#4 (DrpZ3-43C-B) Male	
	allele 1	allele 2
Mafa-F	F-like4	F-like7
Mafa-A	A1*052:02<br>A4*01:04	A1*004:01
Mafa-E	E-like5<br>E-like11	E-like9
Mafa-B	B*095:01<br>B*033:02<br>B*098:10	B*095:01<br>B*033:02<br>B*098:10
	I*01:11	I*01:11
Mafa-DRB	DRB1*10:07<br>DRB1*03:21	DRB1*10:07<br>DRB1*03:21
Mafa-DQA1	DQA1*01:07:01	DQA1*26:03
Mafa-DQB1	DQB1*06:08	DQB1*18:07:02
Mafa-DPA1	DPA1*02:05	DPA1*02:15:02
Mafa-DPB1	DPB1*15:04	DPB1*10:01

Exepriment with the donor cells with HT4 homozygote

	Donor (DrpZ5-32B-C) Male	
	allele 1	allele 2
Mafa-F	F-like1	F-like4
Mafa-A	A1*089:03<br>A2*05:50<br>A3*13:03:01	A1*089:03<br>A2*05:50<br>A3*13:03:01
Mafa-E	E-like3<br>E-like10	E-like3<br>E-like10
Mafa-B	B*104:03<br>B*144:03N<br>B*57:04<br>B*060:02<br>B*046:01:02<br>B*050:08<br>B*114:02<br>B*072:01	B*104:03<br>B*144:03N<br>B*57:04<br>B*060:02<br>B*046:01:02<br>B*050:08<br>B*114:02<br>B*072:01
Mafa-I	I*01:12:01	I*01:12:01
Mafa-DRB	DRB1*10:07<br>DRB1*03:21	DRB1*10:07<br>DRB1*03:21
Mafa-DQA1	DQA1*01:07:01	DQA1*01:07:01
Mafa-DQB1	DQB1*06:08	DQB1*06:08
Mafa-DPA1	DPA1*02:05	DPA1*02:05
Mafa-DPB1	DPB1*15:04	DPB1*15:04

	Cont#5 (2DrpZ5-25C-C) Male	
	allele 1	allele 2
Mafa-F	F-like6	F-like7
Mafa-A	A1*004:01	A1*094:01<br>A2*05:04
Mafa-E	E-like4<br>E-like7	E-like8<br>E-like9
Mafa-B	B*099:01<br>B*108:01<br>B*045:05<br>B*098:08	B*065:03<br>B*161:04<br>B*030:14<br>B*089:01:02
Mafa-I	I*01:10:02	I*01:15:02
Mafa-DRB	DRB*W36:01	DRB1*03:07<br>DRB1*10:06
Mafa-DQA1	DQA1*26:03	DQA1*01:20
Mafa-DQB1	DQB1*18:07	DQB1*06:25
Mafa-DPA1	DPA1*07:04	DPA1*07:02
Mafa-DPB1	DPB1*21:01	DPB1*19:03

	Cont#6 (DrpZ13-47A-G) Male	
	allele 1	allele 2
Mafa-F	F-like2	F-like4
Mafa-A	A1*093:01<br>A1*074:02<br>A8*01:01	A1*038:02<br>A4*14:03
Mafa-E	E-like1	E-like9
Mafa-B	B*076:02<br>B*044:03<br>B*030:05:03	B*056:02:01<br>B*017:01<br>B*157:01<br>B*050:08<br>B*060:03:01<br>B*089:01:02<br>B*116:01
Mafa-I	I*01:26	I*01:11<br>I*01:01:01
Mafa-DRB	DRB3*04:02:01<br>DRB*W1:07<br>DRB*W6:02<br>DRB*W6:03:01	DRB*W53:01<br>DRB*W54:01
Mafa-DQA1	DQA1*24:10	DQA1*01:18
Mafa-DQB1	DQB1*18:26	DQB1*06:35
Mafa-DPA1	DPA1*02:08:02	DPA1*04:02
Mafa-DPB1	DPB1*07:01	DPB1*03:04

	Cont#7 (DrpZ9-81C-A) Male	
	allele 1	allele 2
Mafa-F	F-like6	F-like6
Mafa-A	A1*089:02<br>A2*05:56<br>A3*13:03:01	A1*089:02<br>A2*05:56<br>A3*13:03:01
Mafa-E	E-like9	E-like9
Mafa-B	B*048:03<br>B*041:01<br>B*060:02<br>B*089:01:01	B*137:03
Mafa-I	X	I*01:18:02
Mafa-DRB	DRB*W53:01<br>DRB*W54:01	DRB1*03:17:02<br>DRB1*10:05
Mafa-DQA1	DQA1*01:18	DQA1*24:11
Mafa-DQB1	DQB1*06:35	DQB1*18:27
Mafa-DPA1	DPA1*04:02	DPA1*02:26
Mafa-DPB1	DPB1*03:04	DPB1*08:04

	Cont#8 (2DrpZ4-43C-B) Male	
	allele 1	allele 2
Mafa-F	F-like4	F-like4
Mafa-A	A1*018:04<br>A3*13:03:01	A1*071:02<br>A4*14:14<br>A8*01:01
Mafa-E	E-like3	E-like9
Mafa-B	B*041:01<br>B*101:01:02	B*056:02:01<br>B*017:01<br>B*157:01<br>B*050:08<br>B*060:03:01<br>B*089:01:02<br>B*116:01
Mafa-I	I*01:27	I*01:11<br>I*01:01:01
Mafa-DRB	DRB*W53:01<br>DRB*W54:01	DRB1*03:07<br>DRB1*10:06
Mafa-DQA1	DQA1*01:18	DQA1*01:20
Mafa-DQB1	DQB1*06:35	DQB1*06:25
Mafa-DPA1	DPA1*04:02	DPA1*07:02
Mafa-DPB1	DPB1*03:04	DPB1*19:03

	Hetero#5 (8861E) Male	
	allele 1	allele 2
Mafa-F	F-like1	F-like1
Mafa-A	A1*089:03<br>A2*05:50<br>A3*13:03:01	A1*094:01<br>A2*05:04
Mafa-E	E-like3<br>E-like10	E-like1
Mafa-B	B*104:03<br>B*144:03N<br>B*57:04<br>B*060:02<br>B*046:01:02<br>B*050:08<br>B*114:02<br>B*072:01	B*099:01<br>B*108:01<br>B*045:05<br>B*098:08
Mafa-I	I*01:12:01	I*01:10:02
Mafa-DRB	DRB1*10:07<br>DRB1*03:21	DRB*W53:01<br>DRB*W54:01
Mafa-DQA1	DQA1*01:07:01	DQA1*01:18
Mafa-DQB1	DQB1*06:08	DQB1*06:35
Mafa-DPA1	DPA1*02:05	DPA1*04:02
Mafa-DPB1	DPB1*15:04	DPB1*03:04

	Hetero#6 (DrpZ10-33B-C) Male	
	allele 1	allele 2
Mafa-F	F-like1	F-like4
Mafa-A	A1*089:03<br>A2*05:50<br>A3*13:03:01	A1*018:04<br>A8*01:01
Mafa-E	E-like3<br>E-like10	E-like8<br>E-like9
Mafa-B	B*104:03<br>B*144:03N<br>B*57:04<br>B*060:02<br>B*046:01:02<br>B*050:08<br>B*114:02<br>B*072:01	B*159:01<br>B*007:01:01<br>B*158:01<br>B*085:01<br>B*079:02:02<br>B*098:08
Mafa-I	I*01:12:01	I*01:13:01
Mafa-DRB	DRB1*10:07<br>DRB1*03:21	DRB1*03:07<br>DRB1*10:06
Mafa-DQA1	DQA1*01:07:01	DQA1*01:20
Mafa-DQB1	DQB1*06:08	DQB1*06:25
Mafa-DPA1	DPA1*02:05	DPA1*07:02
Mafa-DPB1	DPB1*15:04	DPB1*19:03

	Hetero#7 (DrpL4-21C-A) Male	
	allele 1	allele 2
Mafa-F	F-like1	F-like6
Mafa-A	A1*089:03<br>A2*05:50<br>A3*13:03:01	A1*089:02<br>A2*05:56<br>A3*13:03:01
Mafa-E	E-like1	E-like9
Mafa-B	B*104:03<br>B*144:03N<br>B*57:04<br>B*060:02<br>B*046:01:02<br>B*050:08<br>B*114:02<br>B*072:01	B*137:03
Mafa-I	I*01:12:01	I*01:18:02
Mafa-DRB	DRB*W1:08<br>DRB*W3:01<br>DRB*W36:01	DRB1*03:17:02<br>DRB1*10:05
Mafa-DQA1	DQA1*26:03	DQA1*24:11
Mafa-DQB1	DQB1*18:07	DQB1*18:27
Mafa-DPA1	DPA1*02:15:02	DPA1*02:15:02
Mafa-DPB1	DPB1*10:01	DPB1*10:01

	Hetero#8 (2DrpZ4-22C-B) Male	
	allele 1	allele 2
Mafa-F	F-like1	F-like6
Mafa-A	A1*089:03<br>A3*13:03:01	A1*038:02<br>A4*14:03
Mafa-E	E-like3<br>E-like10	E-like9
Mafa-B	B*104:03<br>B*144:03N<br>B*57:04<br>B*060:02<br>B*046:01:02<br>B*050:08<br>B*114:02<br>B*072:01	B*099:01<br>B*108:01<br>B*045:05<br>B*098:09
Mafa-I	I*01:12:01	I*01:10:02
Mafa-DRB	DRB3*04:02:01<br>DRB*W6:02<br>DRB*W6:03:01<br>DRB*W1:07	DRB*W33:04<br>DRB1*03:19
Mafa-DQA1	DQA1*24:10	DQA1*24:05
Mafa-DQB1	DQB1*18:26	DQB1*18:05
Mafa-DPA1	DPA1*02:08:02	DPA1*07:04
Mafa-DPB1	DPB1*07:01	DPB1*21:01

Supplementary Table 1| MHC genotypes of two donors and 16 recipient monkeys

MHC alleles derived from HT1 haplotype are highlighted in blue. MHC Class-I alleles derived from HT4 haplotype are highlighted in orange. The same haplotype of MHC Class-II was shared between HT1 and HT4.

Antibodies	Source	Dilution or concentration
For immunocyto- and immunohisto- chemistory		
Nanog	R&D, AF1997	500ng/mL
Oct3/4	SantaCruz, SC5279	500
TubβⅢ	BioLegend, 801201	1000
TH	Millipore, AB152/MAB318	400
Foxa2	R&D, AF2400	500
Lmx1a	gift from KAN Research Institute	500
Nurr1	gift from KAN Research Institute	1000
Ki67	Novo CASTRA,NCL-Ki67p	1000
AIF-1 (Iba-1)	Millipore, ABN67	400
CD45	Dako, M0701	200
HLA-DP, DQ, DR (MHC-classⅡ)	Dako, M0775	500
Monkey IgG-Biotin	Nordic-Mubio BV, GAMon/IgG(H+L)/Bio	200
CD3	Abcam, ab5690	200
CD4	Abcam, ab846	30
CD8	Serotec,MCA609G	200
Girk2	amneolab, APC-006	200
GFP	MBL, 598	500
GFP	Nakalai, 04404-84 (clone GF090R)	1000
NKp46/NCR1	R&D, AF1850	500
For flowcytometery		
PSA-NCAM	Millipore, MAB5324	100
Foxa2-PE	BF, 561589	20
HLA-ABC	BD, 560169	5
HLA-DR	BD, 551375	5

Supplementary Table 2| List of primary antibodies

Gene Name	Forward (5'-to-3')	Reverse (5'-to-3')	Annealing temperature
Mafa-class Ⅰ	TCGTGCGGTTYGAYAGCGACG	CCAGCAYCTCAGGGTGGCCTC	58℃ 45sec, 68℃ 30 sec
Mafa－class Ⅱ DPA1	ATGTTCSAGACCAGAGCT	TTGTCAATGTGGCAGATG	58℃ 45sec, 68℃ 30 sec
Mafa－class Ⅱ DPB1	GCCACTCCAGAGAATTAC	GAGCAGGTTGTGGTGCTG	58℃ 45sec, 68℃ 30 sec
Mafa－class Ⅱ DQA1	ATCCTAAACAAAGCTCTG	TGTGATGTTCACCACAGG	58℃ 45sec, 68℃ 30 sec
Mafa－class Ⅱ DQB1	CTGTGACCTTGATGCTGG	AGACCAGCAGGTTGTGGT	58℃ 45sec, 68℃ 30 sec
Mafa－class Ⅱ DRB	GCTCCCTGGAGGCTCCTG	ACCAGGAGGKTGTGGTGC	58℃ 45sec, 68℃ 30 sec
MAP2	GGATCAACGGAGAGCTGAC	TCAGGACTGCTACAGCCTCA	60℃ 60sec
CORIN	CACAGCCAGGGTCTGGTGGAATGCAG	GAGAGCTACCACCACATGAATCAAGG	60℃ 60sec
FOXA2	ACCCGCTGCAGCCCTTTTGTC	GCAGGTGCTTGAAGAAGCAGGAG	60℃ 60sec
OCT4	GAGAACAATGAGAACCTTCAGGAGA	TTCTGGCGCCGGTTACAGAACCA	58℃ 60sec
GAPDH	GAAGGTGAAGGTCGGAGTC	CATTGATGGCAACAATATCC	60℃ 60sec

Supplementary Table 3| List of primers for quantitative RT-PCR and MHC genotyping
